# Impulsivity, trauma history, and interoceptive awareness contribute to completion of a criminal diversion substance use treatment program for women

**DOI:** 10.3389/fpsyg.2024.1390199

**Published:** 2024-09-04

**Authors:** Emily M. Choquette, Katherine L. Forthman, Namik Kirlic, Jennifer L. Stewart, Mallory J. Cannon, Elisabeth Akeman, Nick McMillan, Micah Mesker, Mimi Tarrasch, Rayus Kuplicki, Martin P. Paulus, Robin L. Aupperle

**Affiliations:** ^1^Laureate Institute for Brain Research, Tulsa, OK, United States; ^2^Department of Community Medicine, University of Tulsa, Tulsa, OK, United States; ^3^Women in Recovery, Family and Children’s Services, Tulsa, OK, United States

**Keywords:** substance use treatment completion, substance abuse treatment, prison diversion program completion, women’s substance use, machine learning

## Abstract

**Introduction:**

In the US, women are one of the fastest-growing segments of the prison population and more than a quarter of women in state prison are incarcerated for drug offenses. Substance use criminal diversion programs can be effective. It may be beneficial to identify individuals who are most likely to complete the program versus terminate early as this can provide information regarding who may need additional or unique programming to improve the likelihood of successful program completion. Prior research investigating prediction of success in these programs has primarily focused on demographic factors in male samples.

**Methods:**

The current study used machine learning (ML) to examine other non-demographic factors related to the likelihood of completing a substance use criminal diversion program for women. A total of 179 women who were enrolled in a criminal diversion program consented and completed neuropsychological, self-report symptom measures, criminal history and demographic surveys at baseline. Model one entered 145 variables into a machine learning (ML) ensemble model, using repeated, nested cross-validation, predicting subsequent graduation versus termination from the program. An identical ML analysis was conducted for model two, in which 34 variables were entered, including the Women’s Risk/Needs Assessment (WRNA).

**Results:**

ML models were unable to predict graduation at an individual level better than chance (AUC = 0.59 [SE = 0.08] and 0.54 [SE = 0.13]). *Post-hoc* analyses indicated measures of impulsivity, trauma history, interoceptive awareness, employment/financial risk, housing safety, antisocial friends, anger/hostility, and WRNA total score and risk scores exhibited medium to large effect sizes in predicting treatment completion (*p* < 0.05; *d*s = 0.29 to 0.81).

**Discussion:**

Results point towards the complexity involved in attempting to predict treatment completion at the individual level but also provide potential targets to inform future research aiming to reduce recidivism.

## 1 Introduction

More than 1.2 million people were incarcerated in US state or federal prisons in 2022 (Carson, 2023). The percentage of women in the prison system has grown from 4% of the population in the 1980s to 7% in 2022 (Carson, 2023). In 2022, 64.6% of women in federal prison were serving time for drug offenses compared to only 44.6% of men (Carson, 2023). Further, while nearly half of US prisoners met criteria for a substance use disorder (SUD), only 33 to 46% of prisoners received treatment (Maruschak et al., 2021). Individuals with SUD are more likely to reoffend and have multiple incarcerations, indicating that SUD treatment is critical for reducing recidivism (Baillargeon et al., 2010; Zgoba et al., 2020). Additionally, non-completion of diversion programs has been linked to higher reoffending rates (Ozturk et al., 2022). Conversely, completion of diversion programs has been linked not only with reduced likelihood of returning to prison (Clifasefi et al., 2017; Bernard et al., 2020; Mueller-Smith and Schnepel, 2021; Ozturk et al., 2022), but also reduced likelihood of substance use overdose (Bernard et al., 2020), reduced cost to state and local governments (Anglin et al., 2013), improved likelihood of being employed (Clifasefi et al., 2017; Mueller-Smith and Schnepel, 2021) and access to stable housed (Clifasefi et al., 2017) with effects lasting up to 20 years (Mueller-Smith and Schnepel, 2021).

In response to overall increases in drug-related incarcerations since the “War on Drugs” in the 1980s (Drug Abuse Office and Treatment Act of 1972, 1972; Bureau of Justice Statistics, 1995; Harvey et al., 2007; Carson, 2023), there are calls for improved access to SUD treatment and alternative programs to divert drug offenders from long-term incarceration (National Institute on Drug Abuse, NIOH, U.S. Department of Health and Human, and Services, 2020; Caulkins et al., 2021). SUD diversion programs often offer reduced or commuted sentences for the completion of a specific program or set of requirements (usually involving SUD treatment) set out for the offender. Outcomes for such programs have been the focus of several review papers, with the focus often being on rate of successful program completion (Hartford et al., 2006; Harvey et al., 2007; Loveland and Boyle, 2007; Sirotich, 2009; Brown R. T., 2010; Brorson et al., 2013; Shapiro et al., 2014; Bernard et al., 2020; Lindquist-Grantz et al., 2021). Rates of completion vary between studies, but reviews suggest dropout rates may be slightly higher for diversion programs at 40–50% (Harvey et al., 2007; Brown R., 2010; Shaffer, 2011) compared to an average of 30.4% across SUD treatment programs types (Lappan et al., 2020).

There is a growing literature examining predictors of successful SUD diversion program completion and recidivism. This literature commonly concentrates on demographic factors including gender (Gray and Saum, 2005; Zettler, 2019; Gallagher et al., 2020), race (Hartley and Phillips, 2001; Gray and Saum, 2005; Ho et al., 2018; Zettler, 2019), and age (Ho et al., 2018; Shannon et al., 2018; Zettler, 2019); and socioeconomic factors such as educational attainment (Hartley and Phillips, 2001; Butzin et al., 2002; Dannerbeck et al., 2006; Ho et al., 2018), employment (Hartley and Phillips, 2001; Butzin et al., 2002; Dannerbeck et al., 2006; Gallagher et al., 2015; Gill, 2016; Ho et al., 2018; Shannon et al., 2018; Zettler, 2019; Gallagher et al., 2020), marital status and social relationships (Dannerbeck et al., 2006; DeVall and Lanier, 2012; Ho et al., 2018), and criminal history (DeVall and Lanier, 2012; Ho et al., 2018; Shannon et al., 2018; Gallagher et al., 2020). These studies suggest that a younger age at time of matriculation, greater extent of criminal history, minority status, less than high school education, and unemployment, may relate to lower likelihood of SUD diversion program completion. However, there are numerous inconsistencies across studies, which may be due to variations in treatment programs and populations – but also likely speaks to the difficulty of predicting mental health and substance use related outcomes including treatment completion (Gowin et al., 2019; Symons et al., 2019).

Research focused on demographic factors predicting treatment outcome is valuable in helping to understand *which individuals* need additional or augmented treatment. However, there have been calls to shift focus to more malleable predictors and to better understand under what conditions dropout occurs, to help inform *what to target* with treatment modifications or augmentations (Zgoba et al., 2020). The current study extends this call to criminal diversion programs in an attempt to understand additional factors related to dropout and increase retention in these programs.

Research focusing on how comorbid mental health and psychological variables relate to program graduation may be particularly useful for identifying SUD treatment augmentation strategies. For instance, comorbid depression symptoms and/or diagnosis at matriculation has been linked to higher rates of attrition from SUD treatment (Curran et al., 2002; Gray and Saum, 2005; Evans et al., 2009; Hickert et al., 2009). Hickert et al. (2009) found that depression in the 30 days prior to treatment more than doubled the likelihood of treatment termination. Evidence for relationships between other comorbid conditions (e.g., anxiety, posttraumatic stress disorder, psychosis) is less clear, but generally supports increased dropout for individuals with dual diagnoses (Levin et al., 2004; Gray and Saum, 2005; Lejuez et al., 2008; Evans et al., 2009; Szafranski et al., 2017; Zettler, 2018). However, it is important to note that some studies suggest comorbid mental illness is *not* associated with termination from diversion or treatment programs (Brown R., 2010; Gallagher et al., 2015) or may increase retention (Amaro et al., 2007; Hesse, 2009; López-Goñi et al., 2021), which also may be due to variations in study populations and treatment programs. For full systematic reviews highlighting some of these inconsistencies see Brorson et al. (2013) and Lappan et al. (2020). Dimensional assessments of behavior or personality have also been explored in relation to treatment completion. Specifically, impulsivity, hostility, aggression, and sensation seeking have been identified as potential predictors of treatment dropout in substance use programs (Loree et al., 2015; Hershberger et al., 2017; Choate et al., 2021). However, one limitation of this previous literature is that it has focused on predominantly male samples. Choate et al. (2021) examined whether gender moderated the relationship between personality traits and treatment termination. They found gender moderated this relationship such that some personality traits (e.g., grandiosity and hostility) related to treatment termination only among men, whereas other traits (e.g., submissiveness) predicted outcomes for women. Further highlighting the need for representation of women in SUD literature is the suggestion that women may have specific needs and barriers to treatment, including lack of services for pregnant women and/or affordable childcare, fear of losing custody, greater transportation concerns, economic concerns, higher rates of comorbid psychiatric conditions, increased likelihood of trauma, greater social stigma and discrimination, and less social support (Greenfield et al., 2007; Tuchman, 2010). While there has been prior work examining gender as a potential predictor of outcome for substance use or criminal diversion programs, further work is warranted to examine factors predicting treatment outcomes and treatment completion for women specifically.

The aim of the current study was to use machine learning (ML) techniques to examine predictors of graduation from a criminal diversion program for women facing SUD related prison sentences. ML is well-suited to this aim because it has the capability to describe complex statistical relationships among high-dimensional data. In such cases, ML has the potential to make better predictions than traditional modeling techniques, improving medical prognoses and diagnostic accuracy (Obermeyer and Emanuel, 2016). The use of ML methods is growing in the SUD literature as a way to examine treatment predictors (Barenholtz et al., 2020); however, it has been less commonly used to examine diversion program completion (Delen et al., 2021). The current study included demographic variables as well as a large battery of psychological variables assessing domains of mental health symptoms, sleep, physical activity, personality, and cognitive functioning. Ultimately, understanding characteristics related to graduation for women enrolled in an SUD criminal diversion program has the potential for informing modification of interventions to increase the likelihood of program completion.

## 2 Method

### 2.1 Participants

Participants were enrolled between November 2015 and October 2018. Rate of study enrollment was influenced by the rate of WIR enrollment combined with the number of women who met inclusion/exclusion criteria for the study. Approximately 315 women enrolled into WIR during the 3 years of study recruitment (approximately 105 per year) of these 304 were screened for the current study and 125 were excluded. The study protocol was designed to be very similar to the Tulsa 1,000 study (Victor et al., 2018) as such inclusion and exclusion criteria were the same with the exception that these participants had to be enrolled in WIR. For full inclusion and exclusion criteria see (Victor et al., 2018). Participants were primarily excluded for psychosis, OCD, or bipolar disorders (*n* = 47). Followed by untreated or complicating medical conditions (e.g., seizure disorders, untreated cancers, history of significant traumatic brain injury, and factors that would impede neuroimaging including high body mass factors that would impede neuroimaging including high body mass; *n* = 43). Additional participants were excluded for pregnancy (*n* = 6), low inclusion scores (*n =* 12), and age (*n* = 1). Reason for exclusion was not listed for 16 participants. Included participants were 179 women (ages 20–55; *m* = 32.8, *SD* = 7.21) enrolled in Women in Recovery (WIR), a court-ordered mental health diversion program for drug-related offenses. Participants were relatively diverse with a slight majority (51.7%) identifying as Non-Hispanic, White (*n* = 91), followed by 29.5% Native American, 8% Black, and 7.4% Hispanic. The most identified drugs of choice were stimulants (55.7%, *n =* 98) and opiates (22.7%, *n =* 40). A total of 130 women (74%) graduated from the program (*n =* 46, 26% terminated). Participants who complete the program receive deferred or dismissed sentences, whereas participants who do not complete are sentenced by the Oklahoma Department of Corrections (DOC). Average time to graduation was approximately 17 months (current sample *m_days_* = 526.39, *SD =* 172.44). See Table 1 for full demographic information.

**Table 1:** Demographic characteristics of full sample by graduation status.

	Graduated (*N* = 130)	Not Graduated (*N* = 46)	Total (*N* = 176)	*p*
Drug of choice				0.218
Opiates	27 (20.8%)	13 (28.3%)	40 (22.7%)	
Other	32 (24.6%)	6 (13.0%)	38 (21.6%)	
Stimulants	71 (54.6%)	27 (58.7%)	98 (55.7%)	
Age (years)				0.042
Mean (SD)	33.4 (7.05)	30.9 (7.41)	32.8 (7.21)	
Median [Min, Max]	31.8 [20.3, 54.2]	29.8 [20.0, 48.1]	31.5 [20.0, 54.2]	
Race/Ethnicity				0.366
Asian	1 (0.8%)	0 (0%)	1 (0.6%)	
Black	7 (5.4%)	7 (15.2%)	14 (8.0%)	
Hispanic	11 (8.5%)	2 (4.3%)	13 (7.4%)	
Native American	39 (30.0%)	13 (28.3%)	52 (29.5%)	
Other	4 (3.1%)	1 (2.2%)	5 (2.8%)	
White	68 (52.3%)	23 (50.0%)	91 (51.7%)	
Education				0.915
No HS	39 (30.0%)	16 (34.8%)	55 (31.3%)	
HS	43 (33.1%)	13 (28.3%)	56 (31.8%)	
Some College	32 (24.6%)	11 (23.9%)	43 (24.4%)	
College or Higher	16 (12.3%)	6 (13.0%)	22 (12.5%)	
85% accrual				1
Mean (SD)	0.186 (0.391)	0.178 (0.387)	0.184 (0.389)	
Median [Min, Max]	0 [0, 1.00]	0 [0, 1.00]	0 [0, 1.00]	
Missing	1 (0.8%)	1 (2.2%)	2 (1.1%)	
N prior probation				0.682
Mean (SD)	0.515 (0.502)	0.565 (0.501)	0.528 (0.501)	
Median [Min, Max]	1.00 [0, 1.00]	1.00 [0, 1.00]	1.00 [0, 1.00]	
Controlling charge category				0.913
Drug	35 (26.9%)	12 (26.1%)	47 (26.7%)	
DUI	6 (4.6%)	1 (2.2%)	7 (4.0%)	
Property	50 (38.5%)	17 (37.0%)	67 (38.1%)	
Violent*	35 (26.9%)	13 (28.3%)	48 (27.3%)	
Missing	4 (3.1%)	3 (6.5%)	7 (4.0%)	

CDDR, customary drinking and drug use record; SD, standard deviation; No HS, no high school diploma or GED awarded; HS, earned high school diploma/GED; 85% Accrual, an 85% charge based on Oklahoma Statute. This indicates that 85% of a sentence must be served due to nature of the crime.*The label of violent crime was made in accordance with Oklahoma Statutes, Title 57; child neglect was the most frequently cited violent crime (33%, *n* = 16); see Supplementary Table 1 for full list of charges included in the Violent category.

The women were recruited for the current study during their first 3 months of beginning the WIR program. Baseline data collected from WIR participants has been combined with the Tulsa 1,000 study for prior cross-sectional analysis studies relating to substance use (Aupperle et al., 2020; Smith et al., 2020a,b, 2021; Stewart et al., 2020). For recruitment research staff from Laureate Institute for Brain Research (LIBR) went to the WIR site on a weekly basis. Any women who were interested in participating could come and complete screening with study staff during these times. With the exception of the Women’s Risk/Needs Assessment (WRNA; Van Voorhis et al., 2010), all measures described were completed by LIBR research staff either on-site at WIR or at LIBR, based on what was most convenient for the participant at the time of assessment. Information provided during study assessments was kept confidential within LIBR study staff, including not being shared with WIR staff or court officials. The WRNA was completed by WIR clinical staff; participants provided consent for this information to be shared with LIBR study staff for the purposes of the research study.

The study was approved by the Western Institutional Review Board. All participants provided written informed consent, in accordance with the Declaration of Helsinki. Additional precautions were taken to decrease potential coercion including individual discussions with each participant by research staff placing an emphasis on the voluntary nature of the study and that their choice to participate or not in the study would have no impact on their treatment at WIR or other correctional facilities. Further, no identifiable data was shared with WIR or the court. Women were compensated for their participation at the rate of $20 per hour via ClinCard^1^, a prepaid debit card used commonly used in clinical trials research.

Controlling charges were determined by the level of security the Oklahoma DOC mandates for an individual based on a conviction for the charge. If an individual had multiple charges the charge with the highest level of security was designated as the controlling charge. The most frequent controlling charge category was Property charges (38.7%, *n =* 67; e.g., prohibited carry of a firearm, carrying a weapon/drug/alcohol into jail). Women fell into four controlling charge categories: Property, Violent, Drug, and Driving Under the Influence (DUI). Table 2 lists the frequency of all criminal charges, wherein the most frequently occurring charges were Unlawful Possession of Paraphernalia (40.3%, *n* = 71), Possession of Controlled Substance (39.2%, *n* = 69), and Larceny of Merchandise from Retailer (23.9%, *n* = 42).

**Table 2:** Description of full sample charges at admission.

Charge	*n*	Percentage
Unlawful possession of paraphernalia	71	40.3%
Possession of controlled substance	69	39.2%
Larceny of merch from retailer	42	23.9%
Distribution of controlled dangerous substance/possession with intent	35	19.9%
Receipt/possession/conceal stolen property $1,000	32	18.2%
Uttering forged instruments	27	15.3%
Possession of controlled dangerous substance (meth) AFCF	25	14.2%
DUI – liquor or drugs/actual physical control in vehicle	21	11.9%
Trafficking in illegal drugs	21	11.9%
Unauthorized use of a vehicle	20	11.4%
Obstructing officer	19	10.8%
Receipt/possession/conceal stolen vehicle	19	10.8%
Child neglect	18	10.2%
Possession of firearm after former conviction of a felony	17	9.7%
Burglary – second degree	13	7.4%
False personation	12	6.8%
Larceny – auto aircraft or other motor vehicle	12	6.8%
Carrying weapon/drugs/alcohol into jail	11	6.3%
Child endangerment	9	5.1%
Robbery or attempted with dangerous weapon	9	5.1%
Careless driving	8	4.5%
Failure to display tax stamp on controlled dangerous substance	8	4.5%
Acquire proceeds from drug activity	7	4.0%
Assault and battery	6	3.4%
Burglary – first degree	6	3.4%
Possession of controlled dangerous substance (Marijuana) AFCF	6	3.4%
Conjoint robbery	5	2.8%
Conspiracy/attempt/endeavor to commit drug crime	5	2.8%
Failure to carry security verification	5	2.8%
False declaration of ownership in pawn shop	5	2.8%
False pretenses, bogus check, or confidence game over $1,000	5	2.8%
Joyriding, loitering in, injuring, or molesting automobile or motor vehicle	5	2.8%
Possession controlled dangerous substance 1000’ school/park/child	5	2.8%
Using offensive weapon in felony	5	2.8%
Assault and/or battery with dangerous weapon	4	2.3%
Assault and battery on a police officer	4	2.3%
Defective vehicle	4	2.3%
Driving W/license canceled/suspended/revoked	4	2.3%
Eluding police officer	4	2.3%
Fraudulently obtaining identity of other	4	2.3%
Make/sell/possession/display false identification	4	2.3%
Possession of credit card belonging to another	4	2.3%
Conspiracy	3	1.7%
Domestic abuse	3	1.7%
Failure to maintain security	3	1.7%
Failure to signal on turning	3	1.7%
False pretenses; trick or deception	3	1.7%
Kidnapping	3	1.7%
Leaving scene of accident involving damage	3	1.7%
Malicious injury/destruction of property	3	1.7%
Obtain or attempt controlled dangerous substance by forgery/fraud	3	1.7%
Public drunk/intoxication	3	1.7%
Trespassing after being forbidden	3	1.7%
Destroying evidence	2	1.1%
Embezzlement	2	1.1%
Escape after lawful arrest	2	1.1%
Failure to yield/turning left	2	1.1%
False pretenses, bogus check, or confidence game under $50	2	1.1%
Grand larceny	2	1.1%
Misuse of forged/counterfeit/suspended driver’s license	2	1.1%
Obtaining property or sign under false pretenses	2	1.1%
Operating vehicle without proper tag/decal	2	1.1%
Personal injury accident while DUI	2	1.1%
Petit larceny	2	1.1%
Possession of forged evidences of debt	2	1.1%
Possession of other forged instruments	2	1.1%
Robbery second degree	2	1.1%
Tamper with surveillance equip to commit crime	2	1.1%
Transporting open container – liquor	2	1.1%
Unauthorized use of credit card	2	1.1%
Unsafe lane use	2	1.1%
Violating security of communications	2	1.1%
Accessory definitions	1	0.6%
Aggravated assault and battery	1	0.6%
Bail jumping	1	0.6%
Contributing to delinquency of minors	1	0.6%
Cultivation of controlled substance	1	0.6%
Escape from confinement	1	0.6%
Failure to stop at stop sign	1	0.6%
Failure to use child restraint system	1	0.6%
Failure to wear seat belt	1	0.6%
Forgery 2nd degree- notes, checks, bills, draft	1	0.6%
Grand larceny from person at night	1	0.6%
Larceny from the house	1	0.6%
Maintaining place for keeping/selling controlled dangerous substance	1	0.6%
No valid drivers license	1	0.6%
Obscene/threatening or harassing phone call	1	0.6%
Possession of counterfeit coin W/intent to circulate	1	0.6%
Possession of sawed-off shotgun/rifle	1	0.6%
Protective order violation	1	0.6%
Receipt/possession/transport stolen copper	1	0.6%
Required position and method of turning	1	0.6%
Transporting open container – beer	1	0.6%
Unlawful use of police radio	1	0.6%

DUI, driving under the influence; AFCF, after former conviction of a felony.

During their enrollment in WIR, women engaged in empirically supported treatment for SUD such as Twelve Step Programs (Kaskutas, 2009) and Seeking Safety (Najavits et al., 1998). As well as for mental health (e.g., individual therapy) including as needed access to trauma/PTSD treatment (e.g., Cognitive Processing Therapy) and pharmacological therapy. Additionally WIR takes a holistic approach to treatment and individuals typically were also enrolled in parenting classes, General Educational Development classes, and occupational assistance as indicated.

### 2.2 Measures

The outcome variable for both models was a binary variable indicating whether or not the participant had completed the WIR program. Within the primary analysis, predictors included 145 clinically relevant measures: demographics, Patient-Reported Outcomes Measurement Information System (PROMIS; Cella et al., 2010), neuropsychological, clinical symptomatology (e.g., positive and negative affect, mental health symptoms), interoception, and personality measures, as well as information collected about criminal charges. For full list of variables see Table 3. The psychological measures were selected to focus on positive and negative valence, cognition, and interoception domains hypothesized to be important transdiagnostic factors important for understanding SUD and other mental health related outcomes. Two indices of criminal behavior/history were included in the analyses: prior probation and 85% crime. Prior probation was entered as a binary variable indicating whether or not the individual had served probation time for their current offense prior to entering the WIR program. Oklahoma Statue has a specific designation for certain criminal offenses that due to the nature of the offense a person shall not serve less than 85% of their imposed sentence. This was coded as a binary variable (Table 2).

**Table 3:** Variables included in the machine learning analysis for model one.

Demographics
Age Race Education Income
Criminal charges
85% crime: based on Oklahoma statute (21 O.S. 13.1) this is a crime for which no less than 85% of the mandatory sentence must be served Prior probation: individual was serving probation or in court custody at time or matriculation
PROMIS measures (Cella et al., 2010)
Social: social abilities, satisfaction with social activities, social isolation, satisfaction with participation in discretionary social activities Positive affect/resilience: positive affect and well-being, emotional support, informational support Negative affect: anger, anxiety, depression Cognitive: applied cognitive abilities, applied general cognitive concerns Sleep: sleep disturbance, sleep-related impairment, fatigue Alcohol: negative expectancies, positive expectancies Nicotine: social motivations, negative psychosocial expectancies, nicotine dependence, negative health expectancies, emotional/sensory expectancies, coping expectancies Pain: physical function, pain interference, pain behavior Sexual interest: interest in sexual activity
Neuropsychological testing
General academic competence: Wide range achievement test-IV (WRAT-IV; Wilkinson, 2006) – Reading: total score Verbal learning and memory California verbal learning test (CVLT; Delis et al., 2000) – False positives, short delay free recall, short delay cued recall, long delay free recall, long delay cued recall, recognition, semantic clustering, total intrusions, total reptations, trial 1 recall, trial 1 to 5 total recall. Information retrieval and executive function Delis-Kaplan executive function system (D-KEFS; Delis et al., 2001) – Verbal fluency: letter, category, category switching, category switching accuracy, repetition errors, set loss errors Attention and working memory Wechsler adult intelligence scale-IV (WAIS-IV; Wechsler, 2008) 7- Digit span: forward, backward, sequencing and total Verbal response inhibition D-KEFS^91^ – Color-word inhibition test: color naming, word reading, naming errors, reading errors, combined reading score, inhibition, inhibition/switching, inhibition errors, inhibition switching errors
Self-reported symptomatology
Physical activity International physical activity questionnaire (IPAQ; Craig et al., 2003): MET minutes (the amount of energy expended carrying out physical activity), and sitting World Health Organization disability assessment schedule (Badu et al., 2021) Self-reported body mass index (BMI) Positive Valence Temporal experience of pleasure scale (TEPS; Gard et al., 2006) – Anticipatory and consummatory scales Negative valence/affect Ruminative responses scale (RRS; Treynor et al., 2003) Overall anxiety severity and impairment scale (OASIS; Norman et al., 2006) State–trait anxiety inventory (STAI; Spielberger et al., 1970)- state and trait scores Anxiety sensitivity index (Taylor et al., 2007) – physical, cognitive, and social subscales and total score Patient health questionnaire (PHQ; Kroenke et al., 2001) - Total Score Positive/negative affect Positive and negative affective schedule expanded form (PANAS-X; Watson and Clark, 1994) Behavioral inhibition/activation scale (BIS/BAS; Carver and White, 1994) – Drive, reward responsiveness, fun seeking and behavioral inhibition scales Eating behaviors SCOFF eating disorder screener (Perry et al., 2002) Three factor eating questionnaire (TFEQ; Stunkard and Messick, 1985) – Cognitive restraint, disinhibition, and hunger scales Eating disorder diagnostic scale (EDDS; Stice et al., 2000) – total score
Alexithymia Toronto alexithymia scale (Taylor et al., 2003) – difficulty describing feelings, difficulty identifying feelings, and externally-oriented thinking subscales and total score Trauma PTSD checklist – civilian version (PCL-C; Conybeare et al., 2012) – total score Traumatic event questionnaire (TEQ; Vrana and Lauterbach, 1994) – total occurrence, total intensity, and worst intensity Childhood trauma questionnaire (CTQ; Bernstein et al., 2003) – physical abuse, sexual abuse, emotional abuse, physical neglect, and emotional neglect, denial subscales, and total score Personality Big five inventory (Whiteside and Lynam, 2001) – extraversion versus introversion, agreeableness versus antagonism conscientiousness versus lack of direction, neuroticism versus emotional stability, and openness versus closedness to experience scales UPPS-P impulsive behavior scale (Whiteside et al., 2005) – lack of premeditation, negative urgency, sensation seeking, and perseverance Interoception Multidimensional assessment of interoceptive awareness (MAIA; Mehling et al., 2012) – noticing, not-distracting, not-worrying, attention regulation, emotional awareness, self-regulation, body listening, and trusting scales Interpersonal reactivity index (IRI; Davis, 1980) – perspective taking, fantasy, empathic concern, and personal distress scales Substance use Drug abuse screening test (DAST; Skinner, 1982) Customary drinking and drug use record (CDDR; Brown et al., 1998) – negative and positive reinforcement Age of onset of primary substance Drug of choice

A secondary analysis was conducted to examine variables from the Women’s Risk/Needs Assessment (WRNA; Van Voorhis et al., 2010). The WRNA is the only validated assessment aimed to determine gender specific risk and needs of women in the criminal justice system (see Table 4 for subscales). The WRNA is completed by WIR staff as part of the clinical intake assessment and the women enrolled in the current study consented to have this intake information shared as part of the research study.

**Table 4:** Variables included in the secondary ML analysis.

Demographics
Age Non-hispanic white
Substance use
Primary substance (simulants, opioids, other) Age of onset of primary substance
Women’s risk/needs assessment (WRNA; Van Voorhis et al., 2010)
Antisocial attitudes Criminal history Educational needs Educational strengths Educational plans Employment/financial Housing safety Antisocial friends Anger/hostility Mental illness score Current psychotropic medications Depression/anxiety symptoms Child abuse Adult abuse Physical abuse Sexual abuse PTSD Substance abuse history Recent substance abuse Significant other Married Children under 18 with contact Family support Family conflict score Relationship difficulties Relationship support Self-efficacy Total risks needs score Total strengths score Total score

### 2.3 Statistical analyses

For the primary analysis, a ML model was used to explore the contribution of 145 unique variables in prediction of graduation status. The R package caretStack (Forthman and Yeh, 2021) was used to train a ML ensemble method which combines the predictions of several ML models for a ‘wisdom of the crowds’ approach (Marbach et al., 2012). We applied three prediction models in this method in order to capture diverse linear and non-linear relationships using methods that are robust to multicollinearity and overfitting: support vector machines (Suykens and Vandewalle, 1999), elastic net (Friedman et al., 2021), and randomForest (Bergstra and Bengio, 2012). Repeated, nested cross-validation (rNCV) has been shown to produce unbiased performance estimates regardless of sample size, making it appropriate for use in the current sample (particularly given that sample size exceeded the number of variables in the model; Vabalas et al., 2019). Optimal hyper-parameter values were chosen using random search (Bergstra and Bengio, 2012), and the one standard error rule (Hastie et al., 2009) using area under the curve (AUC) as the model performance metric. Performance was summarized as the AUC mean and 95% confidence interval. Each prediction model had a different measure provided for variable importance (VI): a “filter” approach (Kuhn, 2021) was used for Support Vector Regression (SVR), absolute values of regression coefficients for elastic net, and an “out-of-bag” mean square error obtained by permutation for randomForest. The VI measures were then scaled between 0 and 100 and overall importance was calculated as the average VI weighted by the performance metric associated with the prediction model. Post-hoc exploratory logistic regressions were conducted between each predictor and graduation status using the glm function in R. Confidence intervals were calculated as two standard errors on the link scale and then the inverse of the link function was applied to map the values back to the response scale. A false discovery rate (FDR) correction was used to correct for multiple comparisons.

For secondary analysis, identical ML and exploratory logistic regressions were conducted but with 34 unique variables (30 subscales from the WRNA, as well as age, race, and substance use variables). The decision was made to not include the WRNA in the primary analysis as the WRNA was only available for a subset of participants (*n* = 125). This was due to the administration of the WRNA. A subsect of the WRNA assessments conducted via paper-and-pencil interviews. Electronic data entry was given priority to WIR graduates which led to a higher graduation rate for this sample.

## 3 Results

### 3.1 Analysis sample

Of the 179 participants recruited for the study, three were excluded for missing the outcome variable (*n* = 2) or having greater than 30% missing data (*n* = 1) for a final analysis sample of *n* = 176 included in the main analysis. Of the 125 participants with data available on the WRNA, one was excluded from the analysis due to missing the outcome variable, leaving a final sample for the WRNA analysis of *n* = 124. The WRNA analysis sample had a higher graduation rate than for women who WRNA data was not available, but there were no other identified demographic differences for those with and without WRNA data available (see Table 5).

**Table 5:** Comparison of full sample and subsample for WRNA analysis.

WRNA record available	FALSE	TRUE	Total	*p*
	(*N* = 53)	(*N* = 124)	(*N* = 177)	
CDDR drug of choice				0.283
Opiates	15 (28.3%)	25 (20.2%)	40 (22.6%)	
Other	8 (15.1%)	30 (24.2%)	38 (21.5%)	
Stimulants	30 (56.6%)	69 (55.6%)	99 (55.9%)	
Age				0.751
Mean (SD)	33.0 (7.40)	32.6 (7.15)	32.7 (7.21)	
Median [Min, Max]	31.7 [20.2, 54.2]	31.4 [20.0, 53.1]	31.5 [20.0, 54.2]	
Race/ethnicity				0.641
Black	6 (11.3%)	8 (6.5%)	14 (7.9%)	
Hispanic	6 (11.3%)	8 (6.5%)	14 (7.9%)	
Native American	14 (26.4%)	38 (30.6%)	52 (29.4%)	
Other	2 (3.8%)	3 (2.4%)	5 (2.8%)	
White	25 (47.2%)	66 (53.2%)	91 (51.4%)	
Asian	0 (0%)	1 (0.8%)	1 (0.6%)	
Education				0.436
College or Higher	5 (9.4%)	17 (13.7%)	22 (12.4%)	
HS	21 (39.6%)	35 (28.2%)	56 (31.6%)	
No HS	14 (26.4%)	42 (33.9%)	56 (31.6%)	
Some College	13 (24.5%)	30 (24.2%)	43 (24.3%)	
Graduated				0.002
Graduated	30 (56.6%)	100 (80.6%)	130 (73.4%)	
Not Graduated	23 (43.4%)	24 (19.4%)	47 (26.6%)	
85% Accrual				0.198
Mean (SD)	0.115 (0.323)	0.211 (0.410)	0.183 (0.388)	
Median [Min, Max]	0 [0, 1.00]	0 [0, 1.00]	0 [0, 1.00]	
Missing	1 (1.9%)	1 (0.8%)	2 (1.1%)	
N prior probation				0.909
Mean (SD)	0.509 (0.505)	0.532 (0.501)	0.525 (0.501)	
Median [Min, Max]	1.00 [0, 1.00]	1.00 [0, 1.00]	1.00 [0, 1.00]	
Controlling charge category				0.885
Drug	15 (28.3%)	33 (26.6%)	48 (27.1%)	
DUI	2 (3.8%)	5 (4.0%)	7 (4.0%)	
Property	21 (39.6%)	46 (37.1%)	67 (37.9%)	
Violent*	12 (22.6%)	36 (29.0%)	48 (27.1%)	
Missing	3 (5.7%)	4 (3.2%)	7 (4.0%)	

CDDR, customary drinking and drug use record; SD, standard deviation; HS, high school education; No HS, less than high school education; 85% Accrual, an 85% charge based on Oklahoma Statute. This indicates that 85% of a sentence must be served due to nature of the crime.*The label of violent crime was made in accordance with Oklahoma Statutes, Title 57; child neglect was the most frequently cited violent crime (33%, *n* = 16); see Supplementary Table 1 for full list of charges included in the Violent category.

All variables of interest had missingness less than 8%. With the exception of age of primary use, which was missing for 31.8% of participants in the full sample.

### 3.2 Machine learning

**Figure 1:**
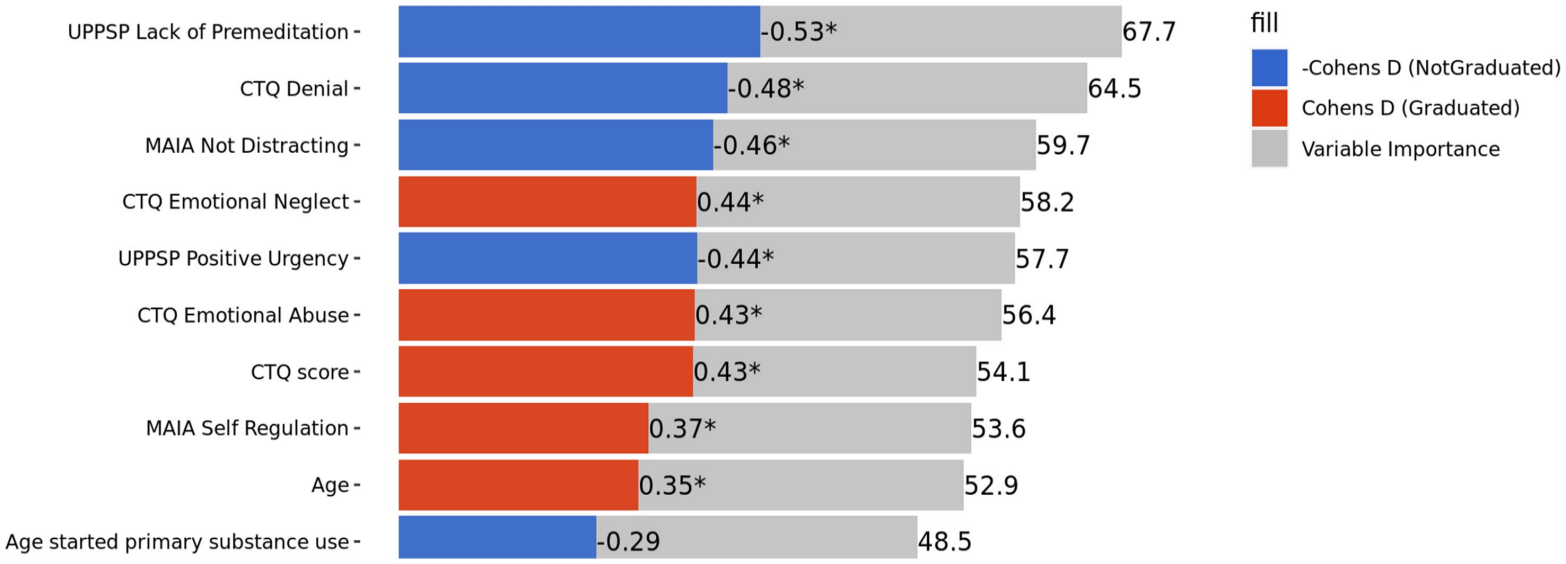
Top 10 variable importance from machine learning model one. “*” Indicates *p*-value <0.05. UPPSP, urgency, premeditation, perseverance, sensation seeking, positive urgency, impulsive behavior scale; CTQ, childhood trauma questionnaire; MAIA, multidimensional assessment of interoceptive awareness.

The stacked model for the primary analysis was conducted examining multidimensional measures of neuropsychological, clinical symptom, interoception, and personality measures (AUC = 0.49; *SE =* 0.08). This was not significantly different from predicting the base rate of completion (see Supplementary Figure S1). Of the variables with the highest variable importance, four were related to trauma (i.e., Childhood Trauma Questionnaire Denial, Emotional Abuse, Emotional Neglect, and Total Score), two were related to impulsivity (UPPS-P Impulsive Behavior Scale Lack of Premeditation and Positive Urgency), and two were related to interoception (Multidimensional Assessment of Interoceptive Awareness Self-Regulation and Not Distracting; see Figure 1). Trauma, self-regulation, and age were positively related to graduation, whereas impulsivity and denial of trauma were negatively related to graduation.

**Figure 2:**
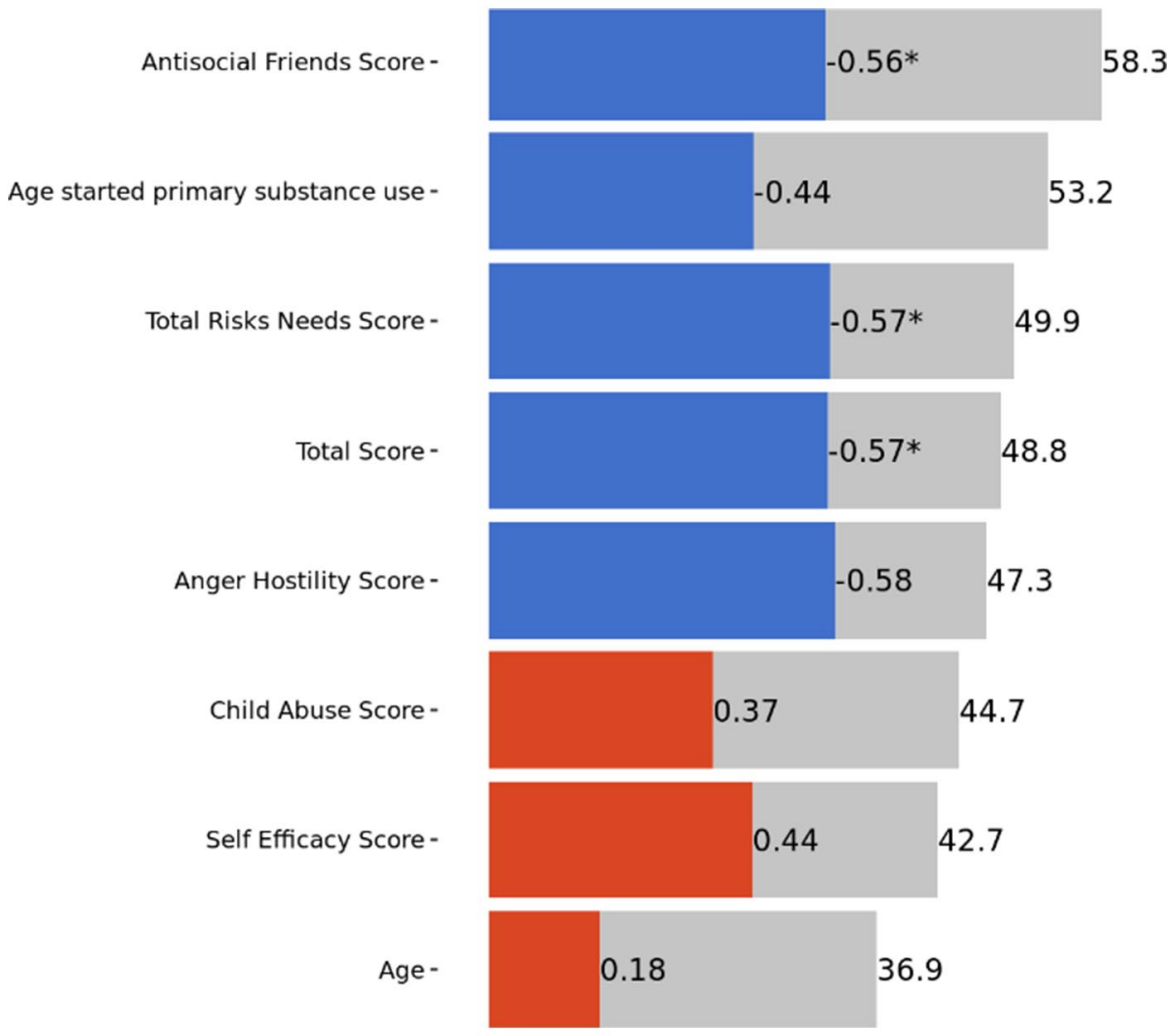
Top 10 variable importance from machine learning model two. “*” Indicates *p*-value <0.05. “***” Indicates significance after Bonferroni correction. Scores were derived from the Women’s Risk/Needs Assessment (WRNA).

When conducting the secondary analysis examining graduation prediction using variables from the WRNA, the model once again was not an improvement on predicting the base rate of completion (see Supplementary Figure S2). The AUC of the stacked model was 0.54 (*SE* = 0.13). Variable Importance was highest for the Employment/Financial score, Housing Safety score, Antisocial Friends score, age of first use of primary substance, Total Risk Needs score, Total score on the WRNA, Anger/Hostility score, Child Abuse score, Self-Efficacy score, and age (see Figure 2). Overall, the top 7 predictors were negatively related to graduation; however, Child Abuse score and Self-Efficacy were positively related to graduation.

### 3.3 *Post-hoc* analyses

**Figure 3:**
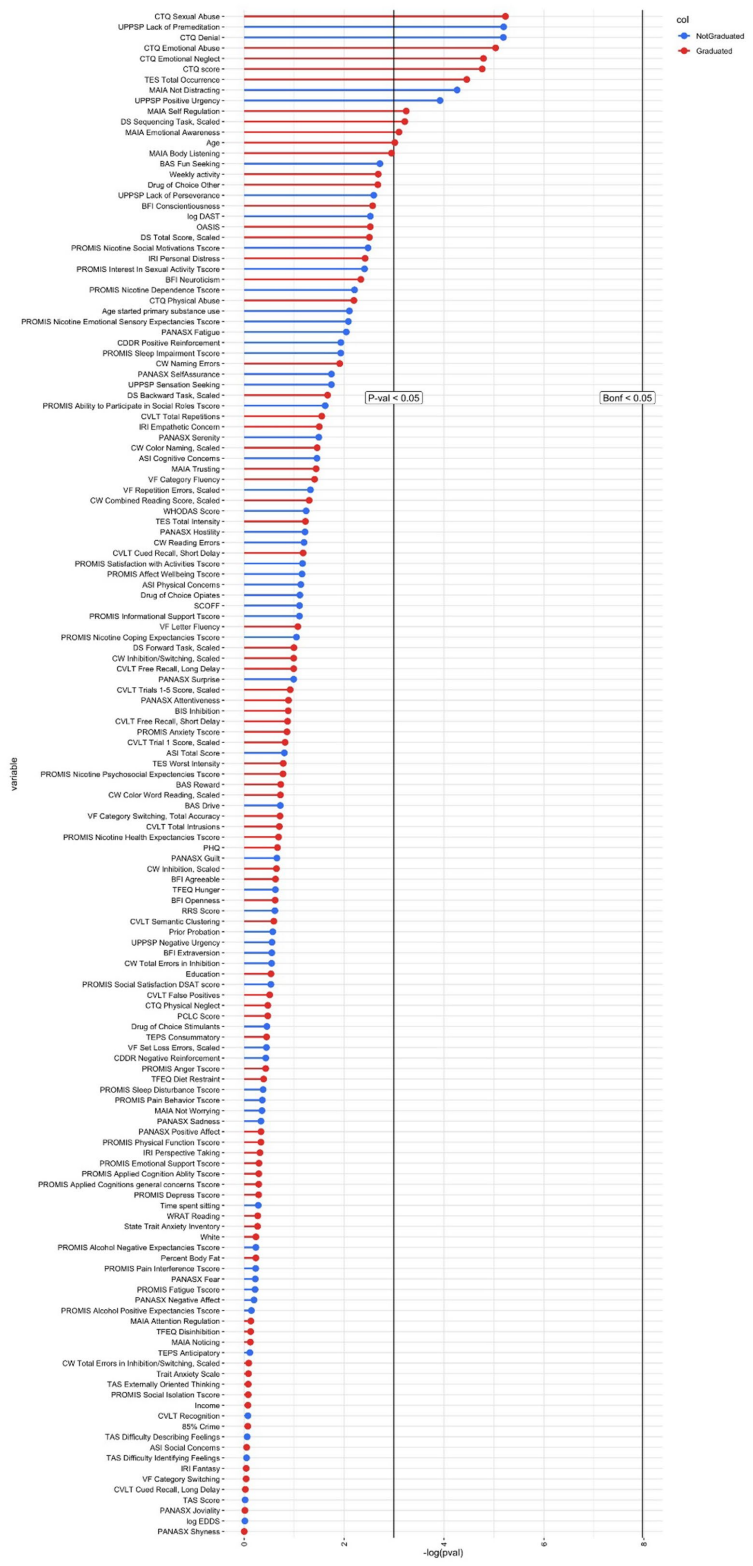
Logistic regressions for variables included in model one. Bonf <0.05 is the threshold for *p*-value significance after Bonferroni correction; red color = negatively related to graduation; blue color = positively related to graduation.

Since ML models were unable to predict graduation better than chance, exploratory logistic regressions were conducted. UPPS-P Lack of Premeditation (*d =* −0.53), CTQ Denial (*d* = −0.48) and MAIA Not Distracting (*d* = 0.46) predicted graduation with medium effect sizes (*p* < 0.05; not significant after FDR correction). See Figure 3.

**Figure 4:**
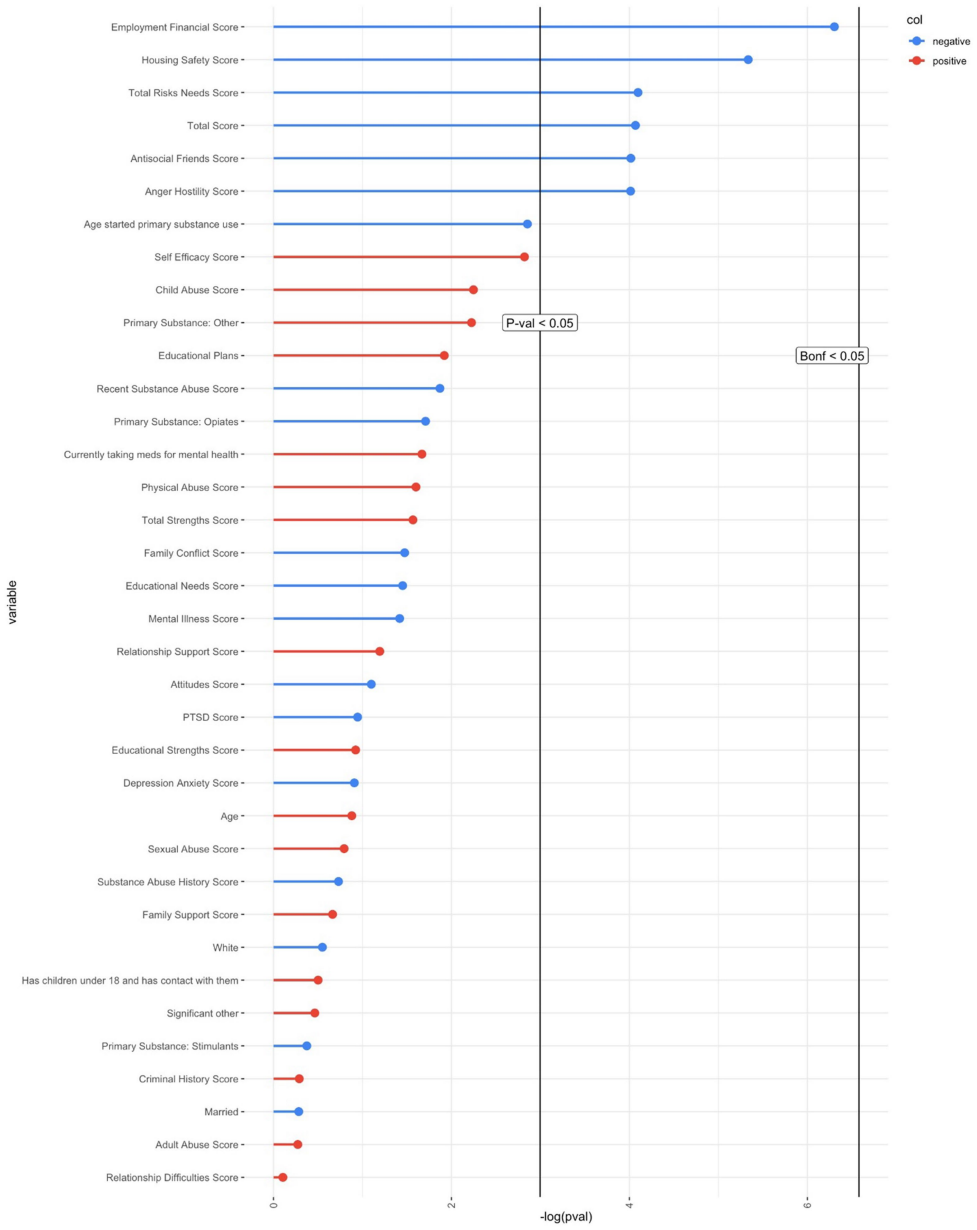
Logistic regressions for variables included in model two. Bonf <0.05 = *p* value corrected using Bonferroni; red color = positively related to graduation; blue color = negatively related to graduation.

The secondary logistic regression models examined the contribution of WRNA variables in predicting graduation status. Employment/ Financial Risk was the only variable that was significant after FDR correction and was negatively related to program completion with a large effect size (Cohen’s *d =* −0.81). Five additional variables had medium to large effect sizes (and were significant at *p* < 0.05) including Housing Safety score (Cohen’s *d =* −0.68), Antisocial Friends score (Cohen’s *d =* −0.56), Anger/Hostility score (Cohen’s *d =* −0.58), Total Risks Needs score (Cohen’s *d =* −0.57), and Total WRNA score (Cohen’s *d =* −0.57). See Figure 4.

## 4 Discussion

The goal of the current investigation was to use data driven techniques to examine predictors of graduation for women participating in an SUD diversion program. Previous work dedicated to identifying predictors of treatment completion often emphasizes the relation between demographic and socioeconomic variables and graduation. This study extends this literature through the inclusion of these variables as well as a large battery of psychological variables assessing domains of mental health symptoms sleep, physical activity, personality, and cognitive functioning. Further, the current study explored graduation in women, a population historically underrepresented in SUD literature. The larger goal was to understand characteristics associated with program completion, with the aspiration to identify strategies for possible intervention or modification of such programs. Unfortunately, even with 147 variables, the ML models were unable to provide individual-level prediction of graduation better than the base rate. However, several variables were identified as being related to graduation with medium to large effect sizes including employment/financial risk, housing safety, anger/hostility, antisocial friends, impulsivity, trauma history, and interoceptive awareness. Many of these identified variables could be potentially targeted in SUD treatment and diversion programs to help improve completion rates.

At the forefront of this discussion, it is important to recognize that the use of ML or other predictive modeling has the potential to perpetuate bias against minoritized populations by relying on factors such as socioeconomic background to make decisions about sentencing (e.g., those sentenced to prison versus criminal diversion programs; Klingele, 2015; DiBenedetto, 2019). We suggest that the identification of such predictors is most beneficial if used to identify potential avenues to enhance treatment retention or success for all individuals enrolled in diversion programs, rather than to identify which individuals to enroll in these programs. ML has been heralded as an approach that may be able to predict at the individual-level mental and substance use related outcomes including treatment completion (Acion et al., 2017; Mak et al., 2019); however, this study and others demonstrate the difficulty of predicting outcomes, even when there is a breadth of information available to include in the models (Gowin et al., 2019; Symons et al., 2019). This may suggest more fine-grained temporal data, such as ecological momentary assessment (Shiffman, 2009), is needed to better predict program completion at the individual level. While our current ML models failed to predict graduation better than chance, the variable importance and logistic regression analyses identified several variables that could potentially be targeted to increase completion rates and may be important to explore in future research. Thus, while the goal of being able to indicate the probability of success of program graduation for any one individual entering treatment remains elusive, the identification of cognitive or behavioral factors relating to treatment completion remain important for informing potential targets for treatment augmentation at the group level (Czajkowski et al., 2015).

In prior work, insight and recognition of one’s problems have been related to increased motivation for treatment, treatment attendance, and abstinence from substances (Raftery et al., 2020). Similarly, in the current study, the CTQ Denial subscale was related negatively to graduation, which may suggest that women who have more insight into their prior experiences of trauma or who are more willing to report trauma, may engage more meaningfully in treatment. Additionally, trauma-informed substance use programs or programs that treat SUD and trauma concurrently have a lower rate of treatment dropout compared to treatment as usual (Amaro et al., 2007; López-Goñi et al., 2021). The current study found trauma to be positively related to graduation, which is seemingly in contradiction to previous studies indicating trauma or trauma-related symptoms relate to worse treatment outcomes (Hyman et al., 2008; Szafranski et al., 2017). This may be related to fact that those who are high in denial are also going to report less trauma experiences and thus, reduced trauma history may instead reflect reduced awareness or willingness to report. However, this finding may also be due to the uniqueness of the current sample. First, the current study examined only women and previous studies recruited majority male samples. Secondly, the women in the current study were recruited from a trauma-informed diversion program. This may have impacted treatment seeking behaviors such that those with significant trauma history may have been more likely to seek out PTSD treatment during WIR. Taken together, findings suggest that insight into one’s mental health concerns and trauma-related care may be important to consider in reducing attrition for women-focused substance use and criminal diversion treatment programs.

The current study also found that UPPS-P Lack of Premeditation was negatively associated with program completion. The UPPS-P Lack of Premeditation subscale assesses one’s tendency to act impulsively without consideration of consequences. A recent meta-analysis found pre-treatment Lack of Premeditation to be a robust predictor of poor therapy outcomes in SUD (Hershberger et al., 2017). Further, this meta-analysis examined pre to post treatment changes in impulsivity and reported a lack of significant change for Lack of Premeditation, Positive Urgency, and Lack of Perseverance and only small effect sizes for the changes in Negative Urgency and Sensation Seeking (Hershberger et al., 2017). Together, these findings suggest that impulsivity is related to treatment completion and that standard substance use treatments may not effectively target impulsivity, indicating potential benefit in identifying strategies that may do so. Various novel treatment augmentation approaches, such as cognitive remediation (Anderson et al., 2021; Nardo et al., 2021) and episodic future thinking (Aonso-Diego et al., 2021; Forster et al., 2021) have shown promise in reducing impulsivity in substance use or other populations. Future research and funding are warranted to identify whether impulsivity interventions in SUD or diversion programs may show promise for better retention and treatment outcomes.

Interoceptive awareness was another psychological construct identified as related to WIR program completion. Increasingly, research has shown a connection between diminished interoceptive awareness and substance use disorder (Smith et al., 2020a; Stewart et al., 2020). The current study suggested that *not* distracting oneself from bodily sensations was related negatively to graduation with a medium effect size, specifically that more distracting increases likelihood of graduating, and interoceptive self-regulation also had a small positive effect size with respect to graduation (Cohen’s *d =* 0.37). Thus, greater awareness of and attempts to regulate interoceptive sensations (via distraction or self-regulation) may be positive indicators for SUD or criminal diversion program completion. Distraction can be both adaptive and maladaptive depending on the context (Wolgast and Lundh, 2017; Waugh et al., 2020), specifically distraction for avoidance is often considered maladaptive; however, distraction for emotion regulation can be adaptive and is considered a useful distress tolerance technique to regulate intense emotions (e.g., as used in dialectical behavioral therapy; Linehan, 2014). It may be that for individuals recovering from SUD, distraction from internal cues, such as cravings, is an adaptive strategy. Treatment strategies that may hold promise for targeting interoceptive awareness and regulation include general distress tolerance and emotion regulation strategies (Linehan, 2014) as well as mind–body interventions, such as mindfulness (Black and Amaro, 2019), yoga (Petker et al., 2021), or Mindful Awareness in Body-Oriented Therapy (Price and Hooven, 2018; Price et al., 2019a,b).

Additional factors which may be useful to address in treatment are employment/finance and housing safety risk factors, which were negatively associated with program completion. While this is the first study to examine the WRNA as it relates to a diversion program for substance-related offences, previous studies have found WRNA scales to be significantly, positively related to subsequent arrests, convictions, and failure to complete probation (Van Voorhis et al., 2010). Additionally, previous studies have found employment to be significantly related to diversion program completion (Dannerbeck et al., 2006; Ho et al., 2018; Shannon et al., 2018). These findings highlight the need to address residential and occupational concerns early in treatment, as doing so may lead to higher rates of program completion.

### 4.1 Limitations

The WIR program is unique in regards to the length of the program and the focus on comprehensive treatment services targeting not only SUD and criminal behavior, but also mental and physical health, and occupational and educational needs. As such, the completion rate of the WIR program (~75%) exceeds that of most SUD or diversion programs in the community and in prior research (Harvey et al., 2007). Thus, generalizability of current findings to other treatment programs is uncertain. It is possible that the high completion rate is due to factors related to selection for the WIR program. Admittance into the WIR program is an extensive process which includes an admissions panel of the WIR clinical directors and must be agreed upon by the district attorney. This admissions panel considers things such as the charges and safety concerns to both the public and treatment milieu. Additionally, participants must be female presenting, have not engaged in this program previously, no psychotic spectrum diagnosis, and at the time of recruitment have charges in Tulsa County. This screening process likely impacted results and may have reduced generalizability. Further, there was a differential rate of graduation in the sample of participants for which the WRNA was available due to preferential data entry for those who graduated. This likely skewed the analyses and could explain some of the null results. Therefore, the results should be interpreted accordingly and replicated in future work. In addition, there may have been factors influencing which women were willing to enroll and met criteria for the research study, which may also limit generalizability. It would be ideal if future studies are conducted using combined data from populations across a variety of diversion programs and numerous sites across the United States. One strength of the current study is the use of longitudinal measurement to predict program completion. Future studies may benefit from assessing changes in some of the domains identified as related to treatment outcome (e.g., impulsivity) to ascertain whether treatment is effectively impacting functioning in these areas. Finally, the study enrollment period was over a 3 year period. While the core content and primary treatments involved in WIR remained similar across the enrollment period, the specific treatment content could have differed across time and participant/provider, given the individualized nature of the WIR program content. These differences could have influenced which measures were identified as predictors of treatment completion.

## 5 Conclusion

This study used a data-driven framework to identify variables contributing to drop out from a women’s substance use criminal diversion program. While the ML model failed to predict graduation rates at the individual level a rate higher than chance, examination of VI and results from logistic regression analyses identified several factors relating to treatment completion with medium to large effect sizes – including trauma, interoceptive awareness, and impulsivity. Future studies are warranted to examine whether interventions aimed at processing trauma or improving interoceptive awareness and impulsivity are related to increased retention in substance use treatment settings.

## Data Availability

The raw data supporting the conclusions of this article will be made available by the authors, without undue reservation.

## Footnotes

^1^ https://www.clincard.com
