# Supplementary figures and images for: Impulsivity, trauma history, and interoceptive awareness contribute to completion of a criminal diversion substance use treatment program for women
Source: Front Psychol. 2024 Sep 4;15:1390199. doi: 10.3389/fpsyg.2024.1390199 (PMC11408307; doi:10.3389/fpsyg.2024.1390199)

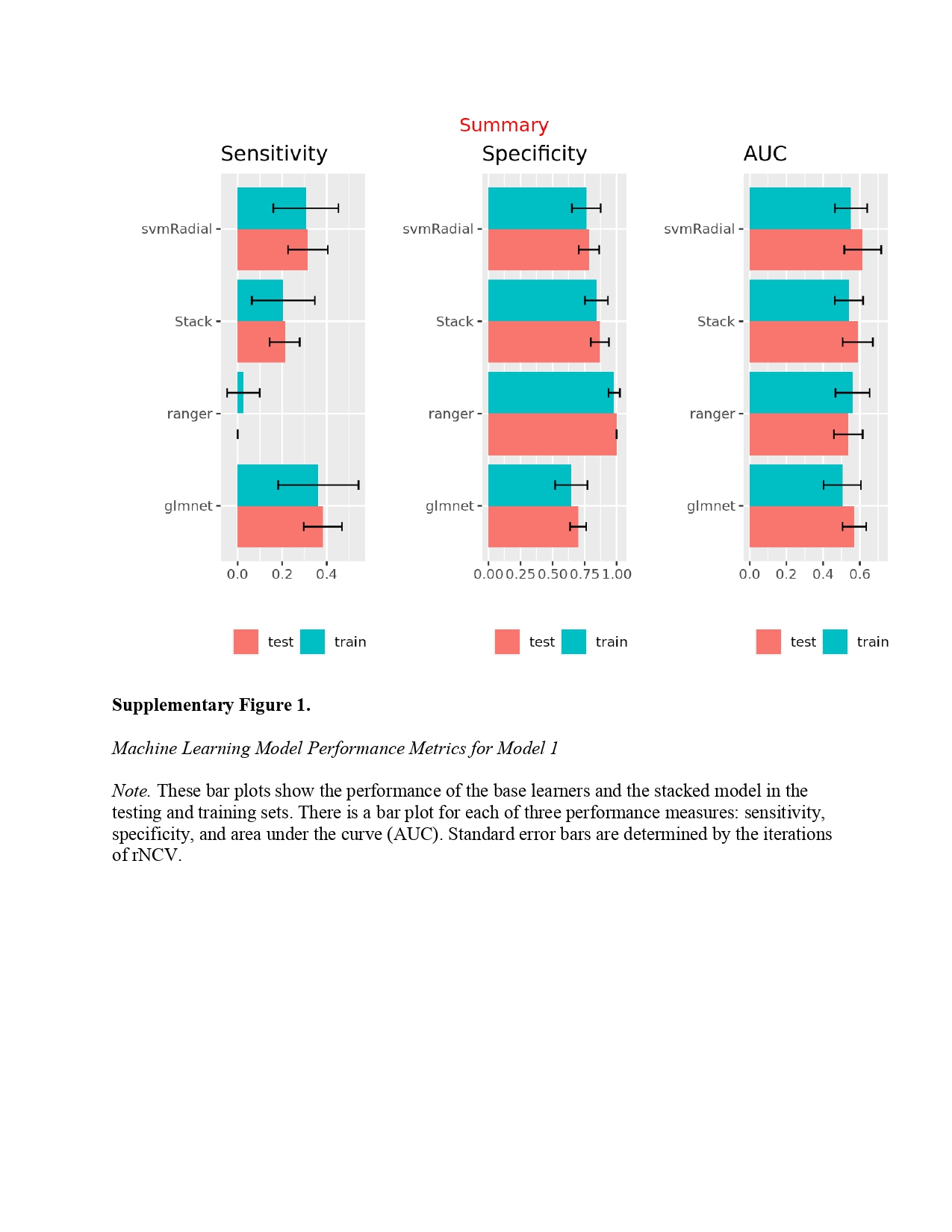

Supplement: Supplementary file 1 [file Image_1.JPEG]

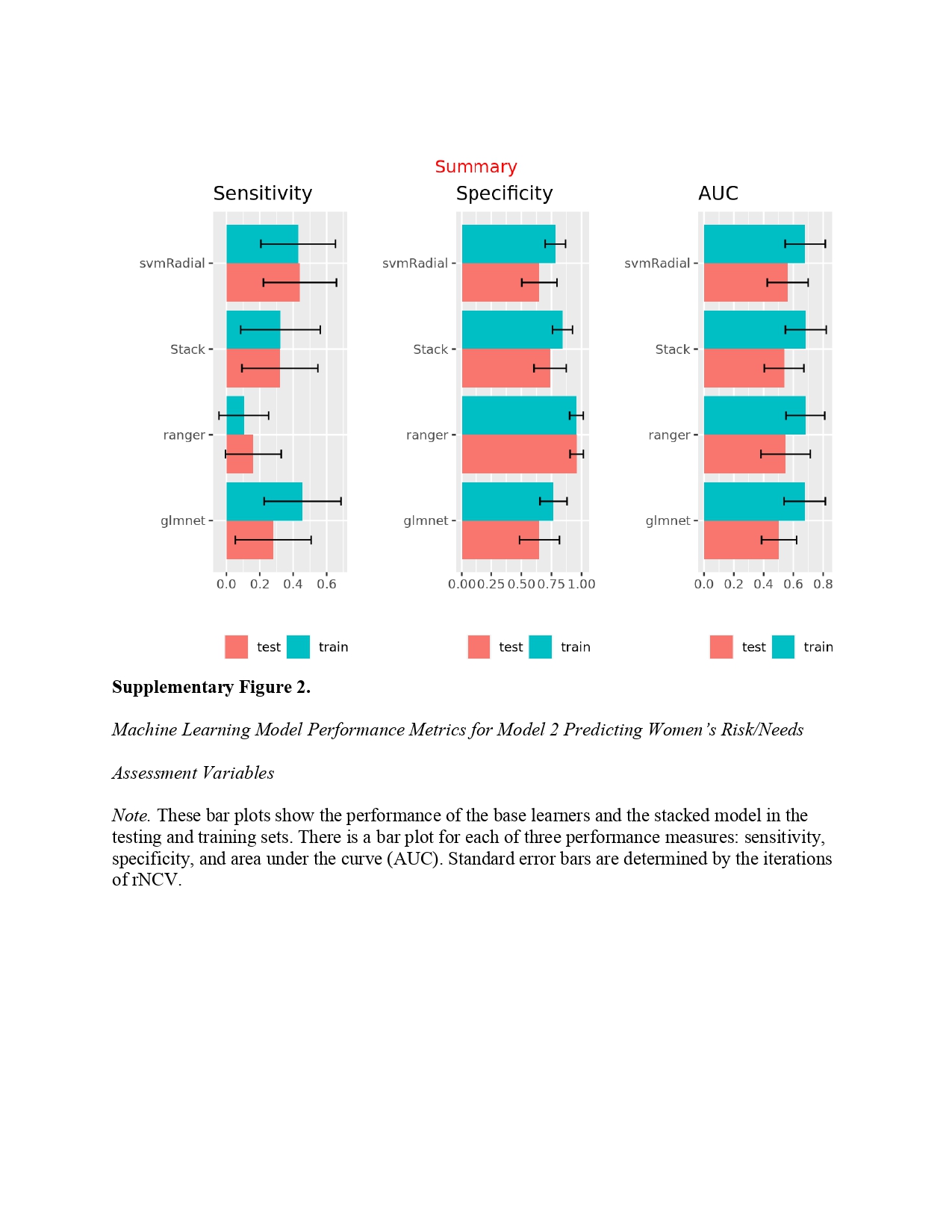

Supplement: Supplementary file 2 [file Image_2.JPEG]
