# Supplementary material for: Impulsivity, trauma history, and interoceptive awareness contribute to completion of a criminal diversion substance use treatment program for women
Source: Front Psychol. 2024 Sep 4;15:1390199. doi: 10.3389/fpsyg.2024.1390199 (PMC11408307; doi:10.3389/fpsyg.2024.1390199)
Supplement: Supplementary file 3 [file Table_1.docx]

| **Supplementary Table 1.**  Description of Crimes Designated as Violent Controlling Charges | | |
| --- | --- | --- |
| Charge | *n* | Percentage |
| Child Neglect | 16 | 33% |
| Robbery Or Attempted W/Dangerous Weapon | 7 | 15% |
| Using Offensive Weapon In Felony | 5 | 10% |
| Conjoint Robbery | 4 | 8% |
| Assault &/Or Battery W/Dangerous Weapon | 3 | 6% |
| Burglary - First Degree | 3 | 6% |
| Kidnapping | 3 | 6% |
| Assault And Battery On A Police Officer | 2 | 4% |
| Robbery Second Degree | 2 | 4% |
| Aggravated Assault And Battery | 1 | 2% |
| Domestic Abuse | 1 | 2% |
| Escape After Lawful Arrest | 1 | 2% |
| *Note.* The label of violent crime was made in accordance with Oklahoma Statutes, Title 57. | | |
